# Evaluation of RNA Secondary Stem-Loop Structures in the UTRs of Mouse Hepatitis Virus as New Therapeutic Targets

**DOI:** 10.3390/pathogens13060518

**Published:** 2024-06-19

**Authors:** Gyuhyun Kang, Sun Hee Lee, Miyeon Cho, Ji-hyeon Kim, Hyosun Cho, Hyojeung Kang

**Affiliations:** 1 Vessel-Organ Interaction Research Center, Research Institute of Pharmaceutical Science, College of Pharmacy, Kyungpook National University, Daegu 41566, Republic of Korea; kgyuhyun14@gmail.com (G.K.); ihappy278@nate.com (S.H.L.); cmy1004g@naver.com (M.C.); 58dekl@naver.com (J.-h.K.); 2 Duksung Innovative Drug Center, College of Pharmacy, Duksung Women’s University, Seoul 01369, Republic of Korea

**Keywords:** MHV-A59, UTR, stem-loop, shRNA, siRNA

## Abstract

MHV-A59 is a beta-coronavirus that causes demyelinating encephalitis and hepatitis in mice. Recently, the mouse infection model of MHV-A59 has been used as an alternative animal infection model for SARS-CoV and SARS-CoV-2, aiding the development of new antiviral drugs. In this study, the MHV-A59 model was employed to investigate the potential of SARS-CoV-2 UTRs as new targets for antiviral drugs. Optimal targets within the MHV-A59 UTRs were identified using a shRNA and siRNA design tool, focusing on RNA secondary stem-loop (SL) structures in the UTRs. We then examined whether the designed RNAi constructs could inhibit MHV-A59 replication. In the 5′UTR, the stem-loop 1 (SL1) was identified as the most effective target, while in the 3′UTR, the minimal element for the initiation of negative-strand RNA synthesis (MIN) proved to be the most effective. Importantly, siRNAs targeting SL1 and MIN structures significantly reduced total RNA synthesis, negative-strand genomic RNA synthesis, subgenomic (sg) RNA synthesis, viral titer, and the plaque size of MHV-A59 compared to the control. Although not statistically significant, the combination of siSL1 and siMIN had a stronger effect on inhibiting MHV-A59 replication than either siRNA monotherapy. Interestingly, while the SL1 structure is present in both MHV and SARS-CoV-2, the MIN structure is unique to MHV. Thus, the SL1 of SARS-CoV-2 may represent a novel and promising target for RNAi-based antiviral drugs.

## 1. Introduction

The Coronavirus family encompasses lethal viruses such as SARS-CoV, MERS-CoV, and SARS-CoV-2, which cause severe respiratory disease in humans [1]. Due to the serious nature of these viruses, high-level biosafety laboratories are essential to ensure the safety of researchers conducting studies on them [2]. The mouse hepatitis virus strain A59 (MHV-A59) was isolated in 1961 from a colony of BALB/c mice, causing leukemia [3]. It was known to infect mice and cause demyelinating encephalitis [4,5]. The principal receptor for the MHV-A59 is the murine carcinoembryonic antigen-related cell adhesion molecule 1 (mCEACAM1) [6,7]. The virus expresses the S protein on the host cell surface by binding to CEACAM-1, enabling it to spread by fusing with neighboring uninfected cells [6].

MHV-A59 is phylogenetically closely related to MERS-CoV, SARS-CoV, and SARS-CoV-2 [2]. In particular, the spike (S), envelope (E), membrane (M), and nucleocapsid (N) proteins of MHV and SARS-CoV-2 share domain features that are unique to coronaviruses. Additionally, MHV and SARS-CoV-2 exhibit similar organotropisms, enabling replication in the nasal respiratory tract, olfactory mucosa, and intestine and facilitating spread to the lungs, liver, and reproductive organs. Both viruses are highly contagious and transmissible by direct contact and respiratory droplets, which are also similar between the two viruses. Finally, the clinical disease, which causes diarrhea, pneumonia, and dehydration, is also partially similar between the two viruses. Consequently, MHV-A59 is regarded as a pivotal model for the investigation of SARS-CoV and SARS-CoV-2, which are highly pathogenic coronaviruses [8].

RNA interference (RNAi) represents a highly effective approach for investigating gene function and has the potential to be employed therapeutically in the treatment of a range of diseases [9]. The two most commonly used RNAi molecules for gene silencing are short hairpin RNA (shRNA) and short interfering RNA (siRNA) [10]. These molecules induce target mRNA degradation through a highly specific and selective mechanism [11]. In the case of delivered shRNA, synthesis occurs in the nucleus of cells, with subsequent transport to the cytoplasm and processing into small siRNA molecules by Dicer [12]. Subsequently, the siRNA is identified by the Argonaute protein complex as the guide strand, which binds to the mRNA through complementary base pairing [13]. This binding occurs at specific sites on the mRNA, resulting in either mRNA degradation or inhibition of translation [12]. The majority of RNAi studies which aimed at inhibiting coronavirus replication targeted coronavirus genes, with only a few targeting the untranslated region (UTR). The sole previous study to target coronavirus UTRs explored the inhibition of replication by randomly selecting a few parts of a specific UTR, but did not screen for effective targets across the UTR [14]. Consequently, we employed the model coronavirus MHV-A59 to screen the entire 5′UTR and 3′UTR in order to identify the optimal RNAi to inhibit MHV-A59 replication.

The genome of MHV-A59 contains untranslated regions (UTRs) at both ends, which form RNA secondary stem-loop (SL) structures through complementary base pairing [15]. Although they do not encode any proteins, these structures play an important role in the replication of the viral genome, the synthesis of negative-strand genomic RNA (gRNA), the synthesis of subgenomic RNA (sgRNA), and the assembly of the viral particle [15,16]. The 5′UTR contains four SLs, designated as stem-loop 1 (SL1), stem-loop 2 (SL2), stem-loop 3 (SL3), and stem-loop 4 (SL4) [17]. The stem region of SL1 contains an U-rich site, which is structurally unstable and crucial for virus replication [18]. The U-rich structure enables the SL to repeatedly unwind and bind with the nearby 3′UTR, thereby maintaining the necessary structure for virus replication. SL2 contains a highly conserved tetra loop sequence, 5′-CUUGU-3′, in members of the Coronaviridae family, and is also involved in sgRNA synthesis [19]. SL3 is a predicted SL structure, as evidenced by the findings of studies 16 and 17. This region contains a transcriptional regulatory sequence (TRS-L), which is involved in the transcription of the virus. SL4 serves as a spacer and is involved in sgRNA synthesis [20]. Given its pivotal role in MHV-A59 replication, sgRNA synthesis, and virion assembly, the SLs represent a promising target for the development of effective inhibitors of MHV-A59 [15,16].

The 3′ cis-acting element for MHV RNA synthesis is located entirely within the MHV genome, spanning nucleotides 31,025 to 31,335. This region comprises three distinct regions [21]. The 3′UTR comprises an essential bulged SL (BSL, nt 31,035~31,102) and an overlapping pseudoknot (PK, nt 31,098~31,151), the hypervariable region (HVR) (nt 31,134~31,289) [22], and the minimal element for the initiation of negative-strand RNA synthesis (MIN) (nt 31,298~31,335) [23]. The BSL and PK are essential for MHV replication and are common to all group 2 coronaviruses. The highly conserved octanucleotide motif, 5′-GGAAGAGC-3′, is present in the HVR and is nonessential for MHV replication. The MIN structure is the minimal signal required for the initiation of negative-strand gRNA synthesis. The 5′ untranslated region (5′UTR) and 3′ untranslated region (3′UTR) are located at both ends of the MHV-A59 genomic RNA, as well as at both ends of each of the six sgRNAs [24]. The shared sequence confers the advantage of simultaneously targeting both the genome and individual sgRNAs, thereby enabling effective targeting across both viral RNA.

Given the pivotal role of the RNA secondary SL structures in MHV-A59 replication, we postulated that RNAi targeting the SLs of UTRs would be an effective means of inhibiting MHV-A59 replication. While numerous studies employing RNAi are currently underway, the majority of these studies focus on the targeting of viral genes, with a paucity of research specifically directed towards UTRs. Consequently, the objective of this research is to identify specific targets within the MHV-A59 UTR SLs that can effectively suppress MHV-A59 replication, thereby contributing to the development of potential therapeutic interventions against coronaviruses.

## 2. Materials and Methods

### 2.1. Virus and Cells

Delayed brain tumor (DBT) cells were maintained at 37 °C and 5% CO_2_ in Dulbecco’s modified Eagle’s medium (DMEM) supplemented with 10% calf serum, 1% streptomycin-penicillin 100× solution (Hyclone, Pittsburgh, PA, USA) and 2% GlutaMAX (Gibco, Waltham, MA, USA). L2 cells were maintained in DMEM supplemented with 10% calf serum, 1% streptomycin-penicillin 100× solution and 2% GlutaMAX at 37 °C and 3% CO_2_. HEK293/T cells were maintained in DMEM supplemented with 10% fetal bovine serum (FBS) (Hyclone), 1% antibiotic-antimycotic 100× solution (Gibco) and 1% GlutaMAX at 37 °C and 5% CO_2_. MHV-A59 was propagated in DBT cells and tittered by plaque assay in L2 cells. The complete genome sequence of MHV-A59 referred to NCBI GenBank (accession number: NC_048217).

### 2.2. Design of MHV-A59 UTR SL Target shRNA and siRNA

We designed four shRNAs, namely shSL1, shSL2, shSL3, and shSL4, referring to each of the four stem-loops within the 5′UTR of MHV-A59. The shRNAs shSL1, shSL2, shSL3, and shSL4 were designed to base-pair with single-stranded RNAs in the loops of SLs 1, 2, 3, and 4 of the 5′UTR, respectively. Furthermore, the full sequences of MHV-A59 5′UTR and 3′UTR were applied to VectorBuilder’s shRNA target design tool (https://www.vectorbuilder.kr/tool/shrna-target-design.html, accessed on 1 January 2022). From the MHV-A59 5′UTR, we designed shSL1/2, shSL3/4, and shSL4, and from the MHV-A59 3′UTR, we designed 3′sh1, 3′sh2, 3′sh3, and 3′sh4 (Supplemental Table S1). To create a hairpin structure, 5′- CCGGTCCGG -sense- CTCTTCAAGAGAGAG -anti sense- TTTTTTGG-3′, the target sequence was inserted into the base of the loop structure in sense and antisense directions. This shRNA structure was used to design the forward primer. The reverse primer was designed in a complementary manner, and *AgeI* and *EcoRI* restriction enzyme sites were added to allow for cloning into the pLKO.1 puro vector. Similar to shRNA design, siRNAs were designed by applying the full sequences of MHV-A59 5′UTR and 3′UTR to GenScript siRNA target finder (https://www.genscript.com/tools/sirna-target-finder, accessed on 1 January 2022). From the MHV-A59 5′UTR, we designed siSL1 and si3/4, and from the MHV-A59 3′UTR, we designed siHVR and siMIN (Supplemental Table S1) [1].

### 2.3. Cloning to Generate Vector-Based shRNA

To generate vector-based shRNA, the designed SL target sequence of MHV-A59 5′UTR was purchased as single-stranded DNA oligonucleotides by Macrogen (Seoul, Korea) (Supplemental Table S1). Each oligonucleotide for shRNA was phosphorylated with T4 Polynucleotide Kinase (NEB, Elpswich, MA, USA) for 1 h and annealing along with complementary oligonucleotide using annealing buffer (10 mM Tris, pH 8.0, 50 mM NaCl, 1 mM EDTA). Equal amounts of complementary oligonucleotides were mixed and heated at 95 °C for 5 min followed by gradual cooling to allow forming of nucleotides duplexes. For vector processing, pLKO.1 vector was digested with restriction enzyme *EcoRI* and *AgeI*. Prepared shRNA cassette and digested pLKO.1 vector was ligated using T4 DNA ligase (Promega, Elpswich, MA, USA) at 16 °C overnight. Ligation products were inserted by electroporation into DH5α competent cells (Thermo Fisher Scientific, Elpswich, MA, USA). Cloned pLKO.1/shRNA plasmids were extracted using a FavorPrep Plasmid Extraction Mini Kit (Favorgen Biotech Corp, Taipei, Taiwan).

### 2.4. Lentivirus Construction

Lentiviruses were produced using envelope and packaging vectors, pMD2.G and pSPAX2, with cloned pLKO.1/shRNA. To generate the lentivirus, pMD2.G (VSV glycoprotein, envelope), psPAX2 (GAG-POL, packaging), and pLKO.1/shRNA were calculated in 4:3:1 molar ratio and mixed in OPTI-MEM media. Vector mixture was co-transfected into HEK293/T cells for 48 h using Lipofectamine 2000 reagent (Invitrogen, Elpswich, MA, USA). The lentivirus supernatants were harvested in conical tube and filtered by 0.2 μm filter. After filtering, lentivirus titrated using qPCR Lentivirus Titer Kit (Applied Biological Materials Inc., Richmond, BC, Canada).

### 2.5. Establishment of shRNA-Treated DBT Cell Line

Lentivirus supernatant with an M.O.I of 1 was used to infect DBT cells for 24 h. Afterward, the lentivirus supernatant was replaced with fresh DME10 medium and treated with 1 μg/mL of puromycin (InvivoGen, San Diego, CA, USA). The medium with 1.0 μg/mL puromycin was replaced every 2 to 3 days until control DBT cells without lentivirus infection were killed by puromycin. The DBT cell lines expressing shRNAs were constructed as follows. First, DBT cells were infected by lentivirus packing shControl vector to construct DBT-shCon cells (Supplemental Table S2). Secondly, lentiviruses packing shRNA SL1 vector, shRNA SL2 vector, shRNA SL3 vector, shRNA SL4 vector, shRNA SL1/2 vector, shRNA SL3/4 vector, shRNA SL4 vector, respectively, infected DBT cells to establish DBT-5′shSL1 cells, DBT-5′shSL2 cells, DBT-5′shSL3 cells, DBT-5′shSL4 cells, DBT-5′shSL1/2 cells, DBT-5′shSL3/4 cells, DBT-5′shSL4′ cells, and DBT-5′shSL1/2 cells, DBT-5’shSL3/4 cells, and DBT-5’shSL4’ cells, respectively (Supplemental Table S2). The cells constructed by infecting DBT cells with a lentivirus packing shRNA targeting the MHV-A59 5’UTR were named DBT-5’shRNA cells to refer to them collectively. Thirdly, lentiviruses packed with 3’sh1 vector, 3’sh2 vector, 3’sh3 vector, and 3’sh4 vector were infected into DBT cells to construct DBT-3’sh1 cells, DBT-3’sh2 cells, DBT-3’sh3 cells, and DBT-3’sh4 cells, respectively (Supplemental Table S2). The cells constructed by infecting DBT cells with lentiviruses packed with shRNA targeting the MHV-A59 3’UTR were collectively referred to as DBT-3′shRNA cells.

### 2.6. siRNA Transfection

A siRNA transfection was performed using RNAiMAX reagent (Thermo Fisher Scientific). DBT cells were seeded at a density of 5 × 10^5^ cells per 6-well plate and incubated at 37 °C with 5% CO_2_. The following day, the media in the dish was replaced with antibiotic-free media. The siRNA was calculated according to the desired concentration for the experiment and added to 250 µL of OPTI-MEM media (Thermo Fisher Scientific), and RNAiMAX reagent was prepared at a concentration of 3.5 µL per well and added to 250 µL of OPTI-MEM media. The mixture was incubated for 5 min. The siRNA and RNAiMAX reagent-containing media were mixed and incubated for 20 min. The prepared mixture was added to each well in a volume of 500 μL. The following day, the media was replaced with complete media containing antibiotics, and after 24 h of transfection, the cells were infected with MHV-A59 for the next experiment. For convenience, DBT cells treated with siSL1 and siSL3/4 were named DBT-5′siSL1 cells and DBT-5′siSL3/4 cells, respectively (Supplemental Table S2). DBT cells treated with siRNA targeting the 5′UTR were collectively named DBT-5′siRNA cells. Next, DBT cells treated with siHVR and siMIN were named DBT-3′siHVR cells and DBT-3′siMIN cells, respectively (Supplemental Table S2). DBT cells treated with siRNA targeting the 3′UTR were collectively named DBT-3′siRNA cells. DBT cells treated with siSL1 and siMIN simultaneously were named DBT-5′3′siRNA cells (Supplemental Table S2).

### 2.7. RNA Dot Blot Analysis

The shRNA- and siRNA-treated DBT cells were prepared as 5 × 10^5^ cells in 6-well plate at 37 °C and 5% CO_2_ for 24 h. MHV-A59 were infected at a multiplicity of infection (M.O.I) 10^−1^ to 10^−5^ in each well for 24 h. Total RNA was extracted using Trizol reagent (Invitrogen). Total RNA sample at concentrations 250 ng/μL and 50 ng/μL were prepared and spotted onto a positively charged nylon membrane (Amersham, Little Chalfont, UK). RNA was linked to the membrane in a UV crosslinker to 254 nm for 2 min. MHV-A59 *N* gene probe was designed using MHV-A59 genome target nucleotide 30,519~31,018 site and made by DIG-labeled DNA probe synthesis kit (Roche, Basel, Switzerland). Prehybridization and hybridization were performed in DIG easy Hyb buffer (Roche) at 50 °C for 16 h. The membrane was then washed at room temperature with low-stringency buffer (2 × SSC, 0.2% SDS) for 5 min and at 50 °C in high stringency buffer (0.1 × SSC, 0.2% SDS) for 15 min. Using DIG wash and block buffer set (Roche), washing and blocking at room temperature for 10 min. For detection of DIG labeled N probe, antibody-digoxigenin-AP (Roche) was diluted 1:20,000 in DIG Blocking buffer and then incubated with membrane at 4 °C, overnight. The membrane was washed using wash buffer for 15 min, twice, and incubated with detection buffer for 3 min. The membrane was incubated with CSP-star solution (Roche) for 3 min and then imaged with LAS-chemiluminescence (Bio-Rad, Hercules, CA, USA). RNA dot blot analysis was performed on DBT cells treated with 5′shRNAs, 5′siRNAs, 3′shRNAs, and 3′siRNAs in at least three replicates each.

### 2.8. Single-Step Growth Curve

In 24-well plate, shRNA- and siRNA-treated DBT cells were cultured to 5 × 10^4^ cells per well at 37 °C and 5% CO_2_ for 24 h. Each well was then infected with MHV-A59 at an M.O.I of 3 and incubated until 0, 6, 12, 18, and 24 h post-infection. At each time point, total virus was collected by scraping the attached cells off with a scraper and collecting them in a conical tube along with the media. The collected MHV-A59 was titrated by plaque assay into L2 cells. For the plaque assay, MHV-A59 collected at each time point was serially diluted 10^−1^ to 10^−6^ using DME0 media. A monolayer of L2 cells was prepared in a 6-well plate and infected with the diluted MHV-A59. After 24 h, L2 cells were fixed using a mixture of 1.6% agarose and 2× DME2 media for 24 h. To observe visible plaques, L2 cells were then fixed with 4% paraformaldehyde (PFA) for 1 h. The gelled media was removed by washing with water, and the 6-well plates were stained with 1% crystal violet in 20% Ethanol (EtOH) for each well. The stained plates were then washed with water and dried overnight. The plaque titer was calculated based on the dilution factor and visualized using GraphPad (GraphPad Prism 5, La Jolla, CA, USA). The plaque size was measured using digimatic calipers (Mitutoyo, Kawasaki, Japan).

### 2.9. Northern Blot Analysis

Northern blot assay was performed to analyze how RNAi targeting the MHV-A59 UTR affects sgRNA production of MHV-A59. The shRNA- and siRNA-treated DBT cells were prepared as 5 × 10^5^ cells in 6-well plate at 37 °C and 5% CO_2_ for 24 h. MHV-A59 were infected at an M.O.I of 0.5 in each well at 24 h. Mock was not infected with MHV-A59 for control RNA sample. Total RNA was extracted using Trizol reagent (Invitrogen). To prevent RNA degradation, immediately after RNA extraction, a 10 µg aliquot of total RNA sample was applied to RNA gel running. Gel electrophoresis was performed on a 1% agarose gel with 7.4% of formaldehyde at 100 V in 1× MOPS buffer (20 mM MOPS, 5 mM sodium acetate, 1 mM EDTA, pH 7.0) for 5 h. Transference onto a positively charged nylon membrane (Amersham) was completed overnight by means of capillary force at room temperature. RNA was linked to the membrane in a UV crosslinker to 254 nm for 2 min. MHV-A59 *N* gene probe was used which was made by DIG-labeled DNA probe synthesis kit (Roche). Prehybridization and hybridization was performed in DIG easy Hyb buffer (Roche) at 50 °C for 16 h. The membrane was then washed at room temperature with low-stringency buffer (2 × SSC, 0.2% SDS) for 5 min and at 50 °C in high stringency buffer (0.1 × SSC, 0.2% SDS) for 15 min. Using DIG wash and block buffer set (Roche), washing and blocking was performed at room temperature for 10 min. For detection of DIG labeled N probe, antibody-digoxigenin-AP (Roche) was diluted 1:20,000 in DIG Blocking buffer and then incubated with membrane at 4 °C, overnight. The membrane was washed using wash buffer for 15 min, twice, and incubated with detection buffer for 3 min. The membrane was incubated with CSP-star solution (Roche) for 3 min and then imaged with LAS-chemiluminescence (Bio-Rad).

### 2.10. 5′ and 3′ Rapid Amplification of cDNA Ends Analysis

To determine whether RNAi targeting the MHV-A59 UTR causes a genomic mutation at the MHV-A59 UTR terminus, we sequenced the PCR products resulting from 5’ Rapid Amplification of cDNA Ends (RACE) and 3’ RACE. The shRNA- and siRNA-treated DBT cells were prepared at 5 × 10^5^ cells per 60-mm dish and infected with MHV-A59 at an M.O.I of 0.5 for 24 h. When cytopathic effects (CPE) were confirmed in the DBT cells, total RNA was extracted using Trizol reagent (Invitrogen) and reverse transcribed using SuperScript III Reverse Transcriptase (Invitrogen) with R1 primer for 5′ RACE assay and AP(dT)17 primer for 3′ RACE assay. The resultant reverse transcription (RT) products were purified using the WIZARD^®^ SV Gel and PCR Clean-Up System (Promega). Poly(A) tailing was then conducted using terminal deoxynucleotidyl transferase (Roche) to add a poly(A) tail to the 3′ ends of the 5′ RACE assay RT products. The poly(A)-tailed 5′ RACE assay RT products were then re-purified. For the first PCR amplification of the poly(A)-tailed 5′ RACE assay RT products, the AP(dT)17 primer and R2 primer were used. The 3′ RACE assay RT products were amplified using the AP(dT)17 primer and F2 primer. For the second PCR, 1 µL of a 1:50 dilution of the previous reaction was used, with the AP(dt)17 primer and R3 primer for 5′ RACE assay products, and the AP(dt)17 primer and F2 primer for 3′ RACE assay products. The final PCR products of both 5′ and 3′ RACE assays were gel-purified and then sequenced by Solgent Analysis Service (Paju, South Korea). The primer sequences used for 5′RACE and 3′RACE are shown in Supplemental Table S3.

### 2.11. Detection of gRNA and sgmRNA

The 5 × 10^5^ DBT cells were seeded in 6-cm culture plates. After 24 h, 20 nM of siCon, siSL1, and siMIN were each transfected into DBT cells. After 24 h, the above DBT cells were infected with MHV-A59 M.O.I 0.5. After another 6 h, total RNAs were extracted from the above DBT cells with RNAeasy kit (QIAGEN, Hilden, Germany). A series of RT-qPCR assays was performed to analyze genomic RNAs (gDNAs) produced by MHV-A59 under siRNA treatments. For analyzing the synthesis of negative-strand gRNA by MHV-A59, the extracted RNAs were primed for reverse transcription by MHV-A59 340-361 sense oligonucleotide (OHK1304), followed by qPCR using MHV-A59 340-361 sense primers (OHK1304) and MHV-A59 410-431 antisense oligonucleotide (OHK1305). For analyzing the synthesis of positive-strand gRNA by MHV-A59, the extracted RNAs were primed for reverse transcription by MHV-A59 768-785 antisense oligonucleotide (OHK1166), followed by qPCR using MHV-A59 340-361 sense primers (OHK1304) and MHV-A59 410-431 antisense oligonucleotide (OHK1305). Actin expression was used as a control. The above extracted RNAs were subjected to reverse transcription with random hexamers, and the resulting cDNAs were subjected to qPCR to quantify actin expression with OHK715 (GGC ATC CTC ACC CTG AAG TA) and OHK716 (GCA CAC GCA GCT CAT TGT AG).

### 2.12. Statistical Analysis

Statistical treatment of two-group data was performed with Student’s *t*-test. Statistical treatment of data from three or more groups was first performed by one-way ANOVA (nonparametric), followed by Tukey’s multiple comparisons test. Statistically significant groups were labeled as A, B, C or A’, B’, C’ to differentiate them. *p*-values < 0.05 (95% confidence) were considered statistically significant. GraphPad Prism version 7.0 (GraphPad Software, San Diego, CA, USA) was utilized for Student’s *t*-test, one-way ANOVA (nonparametric), and Tukey’s multiple comparisons test.

## 3. Results

### 3.1. The Generation of an RNAi-Induced RNA Molecule That Targets the SL of MHV-A59 5′UTR and 3′UTR

The MHV 5’UTR and 3’UTR form an intramolecular RNA secondary structure, which is critical for MHV-A59 replication, specifically for the production of sgRNA and negative-strand gRNA [21,25]. Consequently, we constructed RNAi targeting the MHV-A59 5’UTR and 3’UTR RNA secondary SL structures and analyzed the comparative effectiveness of these RNAi in inhibiting MHV-A59 replication. To target various regions within the 5′UTR and 3’UTR, eleven targets were designed using vector-based shRNA, while four targets were designed using siRNA.

**Figure 1:**
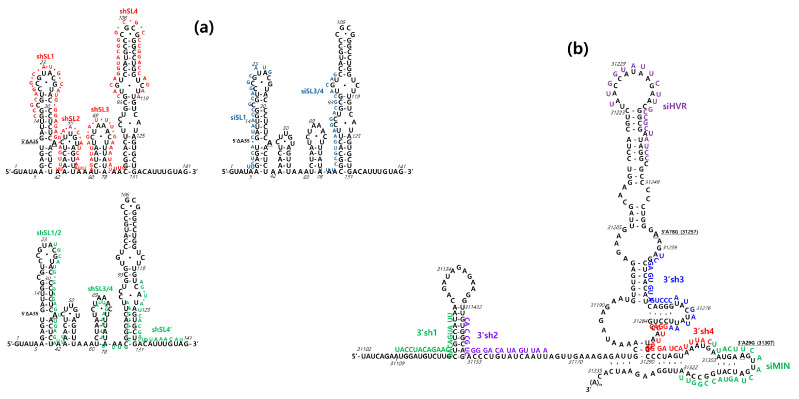
Design of RNAi targets in 5′ and 3′ UTRs in MHV-A59 genome. (**a**) The sequence schematic diagram to display RNAi (shRNA and siRNA) targets in RNA secondary SL structures of MHV-A59 5′ UTR. (**b**) The sequence schematic diagram to display RNAi (shRNA and siRNA) targets in RNA secondary SL structures of MHV-A59 3′ UTR. In a previous study [18], the removal of SL1 A35 (5′ΔA35) in the MHV-A59 5′ untranslated region (UTR) resulted in changes to A31307G (3′A29G, numbered from the 3′ end) and A31257G (3′A78G, numbered from the 3′ end) in the MHV-A59 3′ UTR. The RNA secondary SL structures were labelled 5′ΔA35, 3’A29G, and 3’A78G, respectively.

In the case of shRNAs targeting the MHV-A59 5’UTR, the loop sequence of each SL structure in the 5’UTR was initially employed as the target sequence. For SL1, a 21 bp target sequence (referred to as shSL1) was selected from nt 14~34 of the MHV-A59 genome. For SL2, an 18 bp target sequence (named shSL2) from nt 41~59 was selected. For SL3, a 21 bp target sequence (named as shSL3) from nt 61~81 was selected. For SL4, a 26 bp target sequence (named as shSL4) from nt 94~119 was selected. Secondly, the VectorBuilder shRNA Target Design Tool (https://www.vectorbuilder.kr/tool/shrna-target-design.html, accessed on 1 January 2022) was employed to design effective knockdown targets in the 5′UTR, and the three resulting positions were selected. The selected positions included the overlapping region of SL1 and SL2 from nt 22~44 (referred to as shSL1/2), the overlapping region of SL3 and SL4 from nt 68~88 (referred to as shSL3/4), and the 3′ end position of SL4 from nt 120~140 (referred to as shSL4′). These positions are illustrated in Figure 1a and detailed in Supplemental Table S1.

For shRNAs targeting the MHV-A59 3′UTR, SL sequences in the 3′UTR were employed as the target sequences for the VectorBuilder shRNA Target Design Tool. Two target sequences were selected from the MHV-A59 genome for a pseudoknot in the 3′UTR: a 20 bp target sequence named 3′sh1 from nt 31,109~31,129 and a 19 bp target sequence named 3′sh2 from nt 31,145~31,163. For a terminal SL in the 3′UTR, two target sequences were selected from the MHV-A59 genome: a 21 bp target sequence named as 3′sh3 from nt 31,259~31,280 and a 21 bp target sequence named as 3′sh4 from nt 31,281~31,301 (Figure 1b, Supplemental Table S1).

In the case of siRNA, the GenScript siRNA Design Tool (https://www.genscript.com/tools/sirna-target-finder?page_no = 1&position_no = 2&sensors = googlesearch, accessed on 1 January 2022) was employed to design the siRNAs targeting the MHV-A59 5′UTR and 3′UTR. Two target sequences were selected from the MHV-A59 genome for the 5′UTR SLs: a 21 bp target sequence named as siSL1 from nt 5~25 and a 21 bp target sequence named as siSL3/4 from nt 78~98 (Figure 1a, Supplemental Table S1). For the 3′UTR SLs, two target sequences were selected from the MHV-A59 genome: a 21 bp target sequence named as siHVR from nt 31,225~31,245 and a 21 bp target sequence named as siMIN from nt 31,302~31,322 (Figure 1b, Supplemental Table S1). In order to enhance the silencing efficacy of siRNA, the 3′ end of each siRNA sequence was modified by the addition of two uracils [1]. The extent to which MHV-A59 replication was inhibited was analyzed in DBT cells expressing shRNA or treated with siRNA.

### 3.2. shRNAs Targeting 5′UTR SL Structures Suppress MHV-A59 Replication

**Figure 2:**
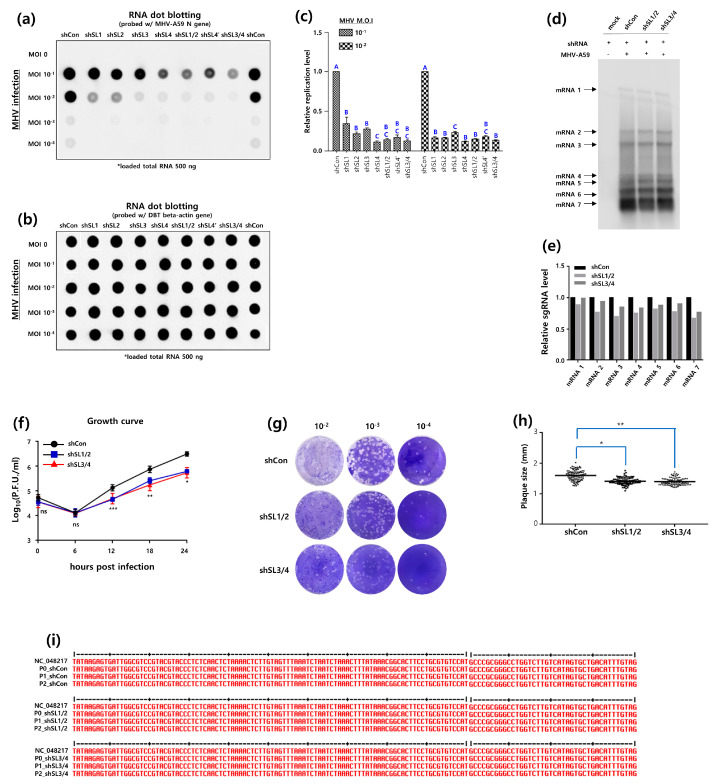
Inhibitory effects of shRNAs targeting RNA secondary SL structures in the 5′UTR on MHV-A59 replication. (**a**) RNA dot blot analysis for inhibitory effects of shRNA on MHV replication. DBT cells (DBT-5′shRNA cells) expressing shRNAs (5′shRNA) targeted for MHV-A59 5′UTR SL structures were established by the shRNA lentiviral system. MHV-A59 was infected at an M.O.I 10^−1^ to 10^−4^ into DBT-5′shRNA cells. RNA dot blot probes were made to detect the MHV *N* gene (NC_048217.1, nt 31,519-31,018). (**b**) As an internal control, the expression of the actin gene (NM_007393.5, nt 668-1180) in DBT-5′shRNA cells was analyzed by RNA dot blot assay. (**c**) The relative inhibitory effect of 5′shRNAs on MHV-A59 infection based on actin expression was quantified by ImageJ software (https://imagej.net/ij/, accessed on 1 January 2023) [27]. One-way analysis of variance was performed, followed by Tukey’s multiple comparison test to verify statistical significance by 5′shRNAs. Statistically significant groups were labeled as A, B, and C to differentiate them. (**d**) Northern analysis for inhibitory effect of 5′shRNA on the MHV sgRNA synthesis. MHV-A59 at an M.O.I of 0.5 was infected into DBT-5′shRNA cells for 24 h. Mock (negative control) was performed with DBT-5′shRNA cells that were not infected with MHV-A59. Patterns of the MHV-A59 sgRNA production in DBT-5′shRNA cells were investigated with a DIG labelled *N* gene probe. (**e**) The effect of 5′shRNA inhibition on the MHV sgRNA synthesis was quantitatively analyzed with Image J software. (**f**) The one-step growth experiments of MHV-A59 infected DBT-5′shRNA cells. 5 × 10^4^ DBT cells were seeded in 24-well plates and infected with MHV-A59 at an M.O.I of 3 for 0, 6, 12, 18, and 24 h the next day. After each infection time, MHV-A59 was harvested and the growth rate of the virus was analyzed. (**g**) Representative plaque shape formed by MHV-A59 infecting DBT-5′shRNA cells. (**h**) Average plaque size of MHV-A59 formed on DBT-5′shRNA cells. Statistical analysis was performed using a student’s *t*-test and a one-way analysis of variance (ANOVA). A post-hoc test was conducted using Tukey’s multiple comparisons test. (**i**) Analysis of the 5′UTR sequence of MHV-A59 infected DBT-5′shRNA cells. The MHV-A59 virus, harvested after infecting DBT-5′shRNA cells, was designated as P0. Subsequently, the harvested virus obtained after re-infecting cells was named P1, and the virus obtained after another round of infection and harvest was named P2. RNA was extracted from each of these virus samples, and sequence analysis was performed. * indicates a statistical difference in the *t*-test between the shCon-treated plaque size group and the shSL1/2 plaque size group; ** indicates a statistical difference in the *t*-test between the shCon-treated plaque size group and the shSL3/4 plaque size group; *** indicates a statistical difference in the *t*-test statistical difference between the shCon-treated plque size group and the siMIN-treated plque size group is significant.

In order to assess the inhibitory effect of shRNA targeting the MHV-A59 5′UTR, DBT cells were transfected with each of the shRNAs, namely shSL1, shSL2, shSL3, shSL4, shSL1/2, sh3/4, and shSL4′ (see Supplementary Table S2). Subsequently, the aforementioned cells were infected with a range of M.O.I. values, spanning from 100 to 10-4 MHV-A59. At 24 h post-infection, viral genome RNA levels were quantified and analyzed. The results demonstrated that the inhibitory effect of shRNAs was highly effective. The viral RNA level at an M.O.I of 10^−1^ appears to have reached its maximum, which may affect the accuracy of relative comparisons. At an M.O.I of 10^−2^, differential effects of shRNAs on the viral RNA detection are evident (Figure 2a,b), rendering it more suitable for data interpretation. With regard to the M.O.I. of 10^−2^, the inhibitory effects of shSL3, shSL4, shSL1/2, shSL4′, and shSL3/4 were found to be relatively satisfactory. It is noteworthy that RNAs targeting shSL3 and shRNA1/2 appear to be effective against high (M.O.I of 10^−2^) and low (M.O.I of 10^−3^) doses of virus infection. In contrast, RNAs targeting shSL1, shSL2, and shRNA4 appear to be less effective. In particular, shSL4 demonstrated a relatively weak inhibitory effect against a M.O.I of 10^−3^, in comparison to higher doses. With regard to the M.O.I of 10^−1^, the inhibitory effect of shSL1/2, sh3/4, and shSL4 was found to be in excess of 85% in comparison to the control (Figure 2c).

As the MHV-A59 genome was found to be significantly reduced in the previous RNA dot blot assay, it was assumed that shRNAs targeting the 5′UTR would also inhibit MHV-A59 sgRNA synthesis. To verify this hypothesis, a Northern blot assay was performed using a probe derived from the MHV-A59 *N* gene. The MHV-A59 genome contains transcription regulatory sequences (TRS), with a leader TRS at the 5′ end of the genome and a body TRS upstream of each open reading frame (ORF) of the sgRNA [26]. The leader TRS forms base pairs with the body TRS, thereby producing the sgRNA. The sgRNAs encode for the spike (S), envelope (E), membrane (M), and nucleocapsid (N) proteins. These proteins are essential for the assembly and budding of the virus. Consequently, the impact of shSL1/2 and shSL3/4 on MHV-A59 sgRNA synthesis was investigated. The synthesis of sgRNA 2 through sgRNA 7 was found to be reduced by shSL1/2 and shSL3/4 (Figure 2d). In particular, shSL1/2 reduced the synthesis of sgRNA 7 by approximately 33% in comparison to the control, while shSL3/4 reduced the synthesis of sgRNA 7 by approximately 23.8% in comparison to the control (Figure 2e).

Given that sgRNA synthesis was reduced in the Northern blot assay, it was postulated that shSL1/2 and shSL3/4 would also affect the life cycle of MHV-A59. To verify this, we conducted a single-step growth experiment of MHV-A59-infected DBT-5′shSL1/2 cells and DBT-5′shSL3/4 cells. At 12 h post-infection, shSL1/2 and shSL3/4 began to decrease the growth rate of MHV-A59, and by 24 h post-infection, this decrease was significant. At 24 h post-infection, shSL1/2 and shSL3/4 showed an 80% decrease in MHV-A59 titer compared to the control (Figure 2f).

Comparative analysis of plaque size is a valuable tool for the relative assessment of viral propagation rates [28]. In general, the faster the virus propagates, the larger the plaque size and the higher the amount of newly produced virus. Consequently, a plaque size analysis was conducted to provide further insight into the results of the one-step growth experiment. The infected DBT-5′shSL1/2 and DBT-5′shSL3/4 cells were harvested and the size of the plaques generated by reinfection of the L2 cells was measured (Figure 2g,h). It is noteworthy that the mean plaque size of MHV-A59-infected DBT-shCon cells was 1.60 mm, while the mean plaque size of MHV-A59-infected DBT-5′shSL1/2 cells and DBT-5′shSL3/4 cells was 1.43 mm and 1.40 mm, respectively (Figure 2h).

In a previous study [18], the removal of SL1 A35 (5′ΔA35) in the MHV-A59 5′ untranslated region (UTR) resulted in changes to A31307G (3′A29G, numbered from the 3′ end) and A31257G (3′A78G, numbered from the 3′ end) in the MHV-A59 3′ UTR (Figure 1). In particular, A31307G (3′A29G) is located in the MIN site. Based on these reciprocal genetic changes, the A35 site in SL1 and the A29 site in MIN were thought to interact with each other. From this perspective, it was hypothesized that the antiviral effect of RNAi targeting the 5′UTR could cause genetic changes in the 3′UTR. Consequently, we conducted genetic sequencing to investigate the genetic changes in the 5′UTR and 3′UTR. In particular, we performed genetic analysis of MHV-A59-infected DBT-5′shSL1/2 cells and DBT-5′shSL3/4 cells in a lineage sequence. The MHV-A59 was harvested from the primary infection of DBT-5′shSL1/2 cells and DBT-5′shSL3/4 cells, and designated as P1_shSL1/2 MHV-A59 and P1_shSL3/4 MHV-A59, respectively. The DBT-5′shSL1/2 cells and DBT-5′shSL3/4 cells were subsequently infected with P1_shSL1/2 MHV-A59 and P1_shSL3/4 MHV-A59, respectively. The harvested MHV-A59 was designated as P2_shSL1/2 MHV-A59 and P2_shSL3/4 MHV-A59. In addition to the control, the 5′UTRs of P1_shSL1/2 MHV-A59, P1_shSL3/4 MHV-A59, P2_shSL1/2 MHV-A59, and P2_shSL3/4 MHV-A59 were sequenced in order to analyze the genetic changes that had occurred. Notably, shSL1/2 and shSL3/4 did not induce any genetic changes in the 5′UTR of MHV-A59 (Figure 2i). For comparison, the 3′UTRs of P1_shSL1/2 MHV-A59, P1_shSL3/4 MHV-A59, P2_shSL1/2 MHV-A59, and P2_shSL3/4 MHV-A59 were also sequenced, and no genetic changes were identified (Supplemental Figure S2). When all the results are taken together, it can be concluded that the knockdown of SL1 and SL2 by shSL1/2 and shSL3/4 severely impairs MHV-A59 replication.

### 3.3. siRNAs Targeting 5′UTR Secondary SL Structures Suppress MHV-A59 Replication

**Figure 3:**
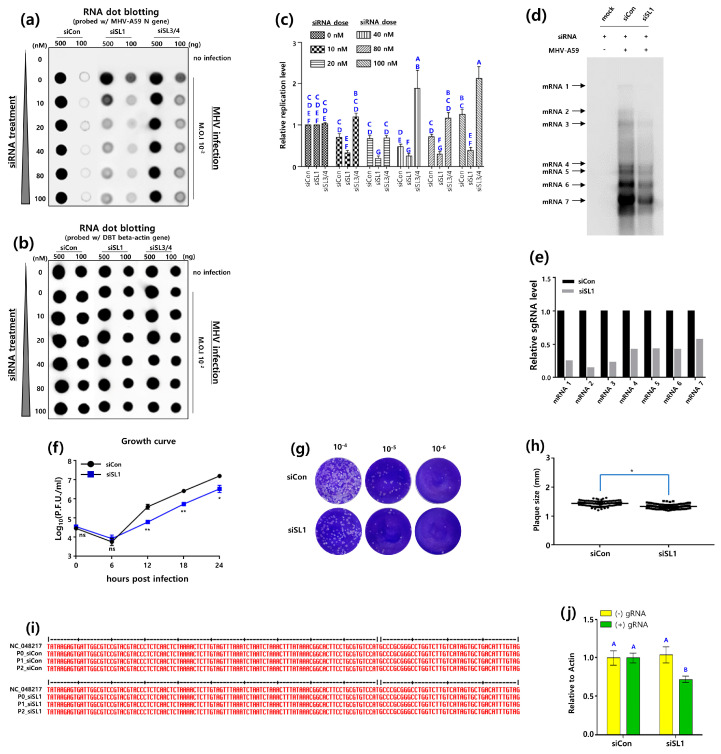
Inhibitory effects of siRNAs targeting RNA secondary SL structures in the UTR on MHV replication. (**a**) RNA dot blot analysis for inhibitory effects of 5′siRNA on MHV replication. DBT cells (DBT-5′siRNA cells) were treated with two concentrations (100, 500 ng) of siRNA (5′siRNA) targeted for MHV-A59 5′UTR SL structures. DBT-5′siRNA cells were infected with MHV-A59 at an M.O.I of 10^−2^ for 24 h. RNA dot blot probes were made to detect the MHV *N* gene. (**b**) As an internal control, the expression of the actin gene in DBT-5′siRNA cells was analyzed by RNA dot blot assay. (**c**) The relative inhibitory effect of siRNAs on MHV-A59 infection based on actin expression was quantified by Image J software. One-way analysis of variance was performed, followed by Tukey’s multiple comparison test to verify statistical significance by 5′siRNAs. Statistically significant groups were labeled as A, B, C, D, E, F and G to differentiate them. (**d**) Northern analysis for inhibitory effect of 20 nM siSL1 on the MHV sgRNA synthesis. MHV-A59 at an M.O.I of 0.5 was infected into DBT-5′siRNA cells for 24 h. Mock (negative control) was performed with DBT-5′siRNA cells that were not infected with MHV-A59. Patterns of MHV-A59 sgRNA production were investigated with a DIG labelled *N* gene probe. (**e**) The effect of 20 nM siSL1 inhibition on the MHV sgRNA synthesis was quantitatively analyzed with Image J software. (**f**) The one-step growth curve of MHV-A59-infected DBT-5′siRNA cells treated with 20 nM siSL1. 5 × 10^4^ DBT cells were seeded in 24-well plates and infected with MHV-A59 at an M.O.I of 3 for 0, 6, 12, 18, and 24 h the next day. After each infection time, MHV-A59 was harvested and the growth rate of the virus was analyzed. (**g**) Representative plaque shape formed by MHV-A59 infecting DBT-siRNA cells treated with 20 nM siSL1. (**h**) Average plaque size of MHV-A59 formed on DBT-5′siRNA cells treated with 20 nM siSL1. Statistical analysis was performed using a student’s *t*-test and a one-way analysis of variance (ANOVA). A post-hoc test was conducted using Tukey’s multiple comparisons test. (**i**) Analysis of the 5′UTR sequence of MHV-A59-infected DBT-5′siRNA cells treated with 20 nM siSL1. The MHV-A59 virus, harvested after infecting DBT-5′siRNA cells, was designated as P0. Subsequently, the harvested virus obtained after re-infecting cells was named P1, and the virus obtained after another round of infection and harvest was named P2. RNA was extracted from each of these virus samples, and sequence analysis was performed. (**j**) Detection of MHV-A59 gRNA in DBT cells treated with 10 nM siSL1. MHV-A59 negative-strand RNA and positive-strand gRNA were quantified relative to actin control by RT-qPCR assay. The negative-strand RNA synthesis in siSL1-treated DBT cells was statistically significantly decreased compared to the negative-strand RNA synthesis in siCon-treated DBT cells. Statistically significant groups were labeled as A and B to differentiate them. * indicates a statistical difference in the *t*-test between the shCon-treated plaque size group and the shSL1/2 plaque size group; ** indicates a statistical difference in the *t*-test between the shCon-treated plaque size group and the shSL3/4 plaque size group.

Subsequently, the RNA dot blot assay was employed to assess the inhibitory impact of the siRNA targeting the MHV-A59 5′UTR. In order to achieve this, we infected DBT-5′siRNA cells (collectively named multiple DBT cells treated with siRNAs targeting the 5′UTR, Supplemental Table S2) with MHV-A59. Subsequently, total RNA was extracted from the aforementioned DBT-5′siRNA cells and subjected to the RNA dot blot assay. The results demonstrated the efficacy of the siRNAs in inhibiting MHV-A59 replication in the DBT cells (Figure 3a,b). The replication of MHV-A59 was reduced by 69% in DBT cells treated with 100 nM siSL1 in comparison to the control, and increased by 68% in DBT cells treated with 100 nM siSL3/4 (Figure 3c). As we observed a reduction of MHV-A59 at the genomic RNA level, we proceeded to examine whether siSL1 could inhibit sgRNA synthesis by utilizing a probe directed to the N protein in the Northern blot assay (Figure 3d). The results demonstrated that siSL1 reduced overall MHV-A59 sgRNA synthesis by approximately 60%, with a specific reduction in the synthesis of mRNA6 by approximately 86% (Figure 3d,e).

Given that the MHV-A59 sgRNA synthesis was reduced in the Northern blot assay, it was hypothesized that siSL1 would also affect the life cycle of MHV-A59. To test this, the single-step growth kinetics of the virus in DBT-5′siSL1 cells were examined for 24 h. The results demonstrated that siSL1 began to reduce the growth rate of MHV-A59 at 12 h post-infection, with a further reduction observed by 24 h post-infection. Furthermore, siSL1 showed an 72.3% decrease in MHV-A59 titer compared to the control at 24 h post-infection (Figure 3f).

Furthermore, the size of the plaques produced by MHV-A59 that infected DBT-5′s iSL1 cells for 24 h each was analyzed. The sizes of the plaques produced by MHV-A59 were analyzed by reinfecting L2 cells with the aforementioned virus (Figure 3g,h). It is noteworthy that the mean plaque size of MHV-A59 infecting DBT-siCon cells was 1.44 mm, while the mean plaque size of MHV-A59 infecting DBT-5′siSL1 cells was 1.33 mm (Figure 3h). In comparison to the siCon control, siSL1 resulted in a reduction in the plaque size of MHV-A59 produced in DBT cells. This reduction in plaque size may be attributed to either a reduction in viral replication or a reduction in viral transmission to neighboring cells.

A two-step sequential passage of MHV-A59 in DBT-5′siSL1 cells was employed to analyze the progeny viruses. The progeny virus derived from the initial infection was designated P1_siSL1, while the progeny virus derived from the second infection was designated P2_siSL1. Sequencing the 5′ untranslated regions (5′UTR) of these progeny viruses revealed that no genetic changes had occurred in the 5′UTR after the two-step sequential passage (Figure 3i). Similarly, sequencing of the 3′UTRs demonstrated that no genetic changes had occurred (Supplemental Figure S2).

In a previous study, Leibowitz et al. reported that the chimeric MHV-A59/SCoV-SL3, which replaced the MHV-A59 SL3 with the SARS-CoV (SCoV) SL3, was able to produce the negative-strand gRNA but failed to produce the positive-strand sgRNA, resulting in a nonviable genome [15]. Based on these findings, we postulated that siSL1 inhibits the synthesis of the negative-strand gRNA of MHV-A59. To test this hypothesis, we analyzed whether gRNA synthesis of MHV-A59 was inhibited in DBT cells treated with siSL1. As illustrated in Figure 3j, the administration of 10 nM siSL1 resulted in a notable inhibition of the synthesis of the MHV-A59 positive-strand gRNA, while the negative-strand gRNA remained unaffected. In conclusion, these results demonstrate that the knockdown of SL1 by siSL1 significantly impairs MHV-A59 replication.

### 3.4. shRNAs Targeting 3′UTR RNA Secondary SL Structures Regulate MHV-A59 Replication

The MHV-A59 3′UTR, which is 301 nt in length, is composed of an RNA secondary structure. The 3′UTR displays a bulged stem-loop (BSL), overlapping pseudoknot (PK), hairpin loop (HVR), and minimal internal structure (MIN) configuration. Consequently, we constructed 3′ short hairpin RNAs (shRNAs) targeting these structures, transfected DBT cells with 3′ shRNAs, and examined their inhibitory effect on MHV-A59 replication by quantifying viral genomic RNA.

**Figure 4:**
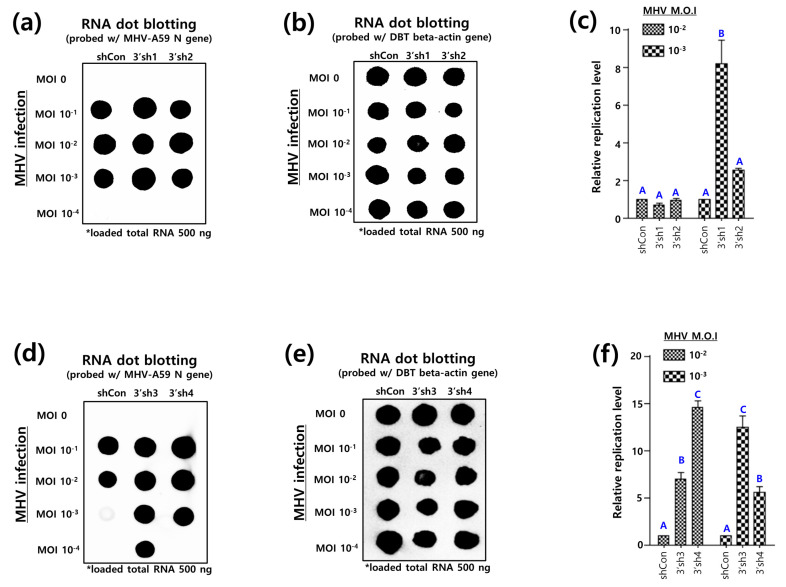

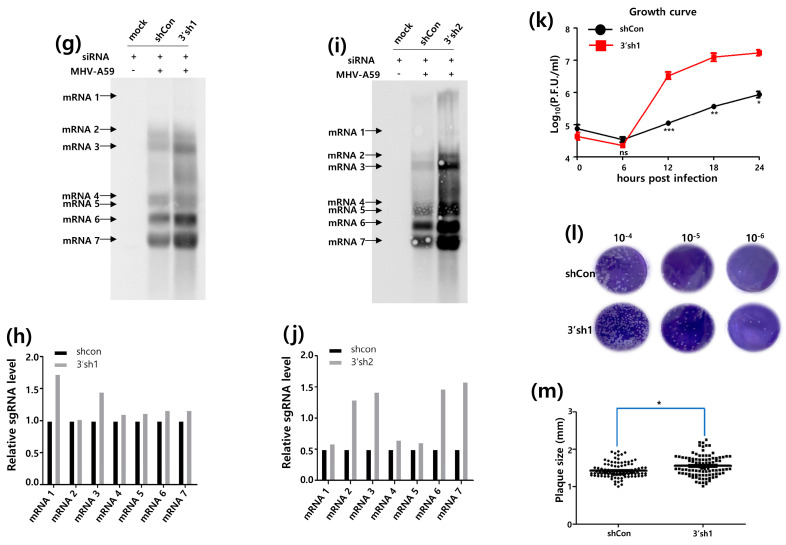
Inhibitory effects of shRNAs targeting RNA secondary SL structures in the 3′UTR on MHV replication. (**a**) RNA dot blot analysis for inhibition of MHV replication by 3′sh1 and 3′sh2 targeting MHV-A59 3′UTR pseudoknot. DBT-3′sh1 and DBT-3′sh2 cells expressing 3′sh1 and 3′sh2 were established by the shRNA lentiviral system. MHV-A59 was infected at an M.O.I of 10^−1^ to 10^−4^ into DBT-3′sh1 and DBT-3′sh2 cells, respectively. RNA dot blot probes were made to detect the MHV *N* gene. (**b**) As an internal control, the expression of the actin gene in DBT-3′sh1 and DBT-3′sh2 cells was analyzed by RNA dot blot assay, respectively. (**c**) The relative inhibitory effect of 3′sh1 and 3′sh2 on MHV-A59 infection based on actin expression was quantified by Image J software, respectively. MHV-A59 *N* gene expression was evaluated relative to actin gene expression in DBT-3′sh1 and DBT-3′sh2 cells infected with MHV-A59, respectively. One-way analysis of variance was performed, followed by Tukey’s multiple comparison test to verify statistical significance by 3′shRNAs. Statistically significant groups were labeled as A and B to differentiate them. (**d**) RNA dot blot analysis for inhibition of MHV replication by 3′sh3 and 3′sh4 targeting MHV-A59 3′UTR pseudoknot. DBT-3′sh3 and DBT-3′sh4 cells expressing 3′sh3 and 3′sh4 were established by the shRNA lentiviral system. MHV-A59 was infected at an M.O.I of 10^−1^ to 10^−4^ into DBT-3′sh3 and DBT-3′sh4 cells, respectively. RNA dot blot probes were made to detect the MHV *N* gene. (**e**) As an internal control, the expression of the actin gene in DBT-3′sh3 and DBT-3′sh4 cells was analyzed by RNA dot blot assay, respectively. (**f**) The relative inhibitory effect of 3′sh3 and 3′sh4 on MHV-A59 infection based on actin expression was quantified by Image J software, respectively. MHV-A59 *N* gene expression was evaluated relative to actin gene expression in DBT-3′sh3 and DBT-3′sh4 cells infected with MHV-A59, respectively. One-way analysis of variance was performed, followed by Tukey’s multiple comparison test to verify statistical significance by 3′shRNAs. Statistically significant groups were labeled as A, B and C to differentiate them. (**g**) Northern analysis for inhibitory effect of 3′sh1 on the MHV sgRNA synthesis. MHV-A59 at an M.O.I of 0.5 was infected into DBT-3′sh1 cells for 24 h. Mock (negative control) was performed with DBT-3′sh1 cells that were not infected with MHV-A59. MHV sgRNA production pattern was probed with a DIG labelled *N* gene probe. (**h**) The effect of 3′sh1 inhibition on MHV sgRNA production was quantitatively analyzed with Image J software. (**i**) Northern analysis for inhibitory effect of 3′sh2 on MHV sgRNA synthesis. MHV-A59 at an M.O.I of 0.5 was infected into DBT-3′sh2 cells for 24 h. Mock (negative control) was performed with DBT-3′sh2 cells that were not infected with MHV-A59. MHV sgRNA synthesis pattern was probed with a DIG labelled *N* gene probe. (**j**) The effect of 3′sh2 inhibition on the MHV sgRNA synthesis was quantitatively analyzed with Image J software. (**k**) The one-step growth experiments of MHV-A59-infected DBT-3′sh1 cells. 5 × 10^4^ DBT cells were seeded in 24-well plates and infected with MHV-A59 at an M.O.I of 3 for 0, 6, 12, 18, and 24 h the next day. After each infection time, MHV-A59 was harvested and the growth rate of the virus was analyzed. (**l**) Representative plaque shape formed by MHV-A59 infecting DBT-3′sh1 cells. (**m**) Average plaque size of MHV-A59 formed on DBT-3′sh1 cells. Statistical analysis was performed using a student’s *t*-test and a one-way analysis of variance (ANOVA). A post-hoc test was conducted using Tukey’s multiple comparisons test. * indicates a statistical difference in the *t*-test between the shCon-treated plaque size group and the shSL1/2 plaque size group; ** indicates a statistical difference in the *t*-test between the shCon-treated plaque size group and the shSL3/4 plaque size group; *** indicates a statistical difference in the *t*-test statistical difference between the shCon-treated plque size group and the siMIN-treated plque size group is significant.

To this end, we infected DBT-3′shRNA cells (collectively designated as multiple DBT cells expressing shRNAs targeting the 3′UTR, Supplemental Table S2) with MHV-A59. Subsequently, total RNA was extracted from the aforementioned DBT-3′shRNA cells and subjected to an RNA dot blot assay. The results demonstrated that the shRNAs did not inhibit, but rather enhanced, MHV-A59 replication in the DBT cells (Figure 4a–d). In DBT-3′shRNA cells infected with M.O.I 10^−3^ MHV-A59, 3′sh1, 3′sh2, 3′sh3, and 3′sh4 demonstrated an 8.1-, 2.5-, 12.4-, and 5.5-fold increase in MHV-A59 replication, respectively, in comparison to the control (Figure 4c,f). Furthermore, DBT-3′sh3 and DBT-3′sh4 cells demonstrated elevated levels of viral replication following M.O.I 10^−2^ MHV-A59 infection, when compared to controls (Figure 4f).

In a similar vein to the aforementioned studies, we conducted a Northern blot assay to ascertain whether 3′shRNA impedes the synthesis of MHV-A59 sgRNA. The Northern blot assay demonstrated that 3′sh1 produced a greater number of sgRNAs compared to the control (Figure 4g,h), while 3′sh2 produced a significantly greater number of sgRNAs compared to the control (Figure 4i,j).

Given that the synthesis of sgRNAs was increased in the Northern blot assay, it was hypothesized that 3′shSL1 would affect the life cycle of MHV-A59. Furthermore, the impact of 3′shSLs on the life cycle of MHV-A59 in DBT-3′shSL1 cells was examined. The growth rate of MHV-A59 was found to be increased in the presence of 3′sh1, compared to the control, from 12 h post-infection onwards, with this increase persisting until 24 h post-infection (Figure 4f).

Furthermore, the size of the plaques produced by MHV-A59 that infected DBT-3′shSL1 cells for 24 h each was analyzed. Plaque size was analyzed by reinfecting L2 cells with the aforementioned MHV-A59 (Figure 4l,m). It is noteworthy that the mean plaque size of MHV-A59 infecting DBT-shCon cells was 1.42 mm, while the mean plaque size of MHV-A59 infecting DBT-3′shSL1 cells was 1.55 mm (Figure 4m). In conclusion, the results demonstrate that the knockdown of the overlapping pseudoknot by 3′shSL1 significantly enhances MHV-A59 replication.

### 3.5. siRNAs Targeting 3′UTR Secondary SL Structures Suppress MHV-A59 Replication

**Figure 5:**
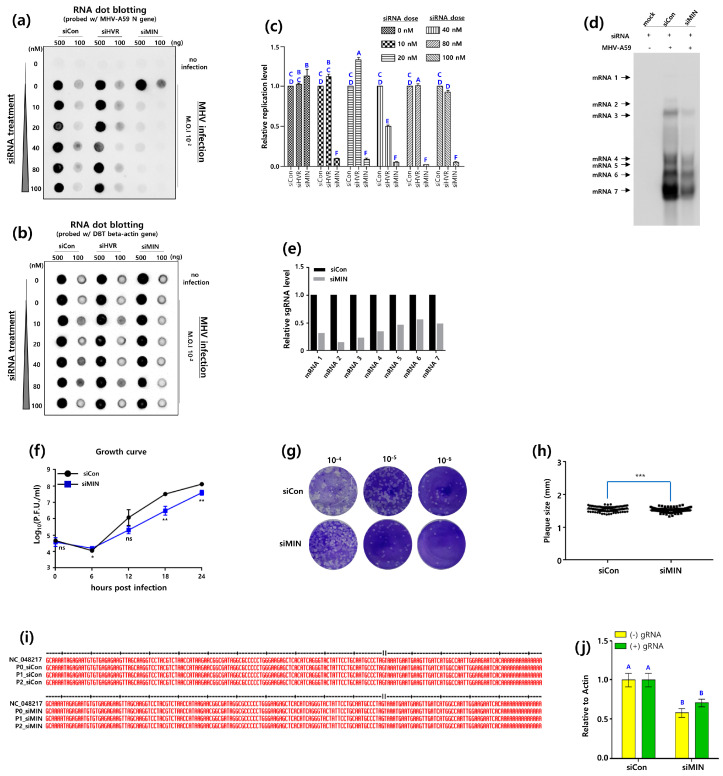
Inhibitory effect of siRNAs targeting RNA secondary SL structures in the 3′UTR on MHV replication. (**a**) RNA dot blot analysis for inhibitory effect of 3′siRNA on MHV replication. DBT cells (DBT-3′siRNA cells) were treated with two concentrations (100, 500 ng) of siRNA (3′siRNA) targeted for MHV-A59 3′UTR SL structures. DBT-3′siRNA cells were infected with MHV-A59 at an M.O.I of 10^−2^ for 24 h. RNA dot blot probes were made to detect the MHV *N* gene. (**b**) As an internal control, the expression of the actin gene in DBT-3′siRNA cells was analyzed by RNA dot blot assay. (**c**) The relative inhibitory effect of 3′siRNAs on MHV-A59 infection based on actin expression was quantified by Image J software. MHV-A59 *N* gene expression was evaluated relative to actin gene expression in DBT-3′siRNA cells infected with MHV-A59. One-way analysis of variance was performed, followed by Tukey’s multiple comparison test to verify statistical significance by 3′siRNAs. Statistically significant groups were labeled as A, B, C, D, E and F to differentiate them. (**d**) Northern analysis for inhibitory effect of 20 nM siMIN on the MHV sgRNA synthesis. MHV-A59 at an M.O.I of 0.5 was infected into DBT-3′siRNA cells for 24 h. Mock (negative control) was performed with DBT-3′siRNA cells that were not infected with MHV-A59. Patterns of MHV-A59 sgRNA production in DBT-3′siRNA cells were investigated with a DIG labelled *N* gene probe. (**e**) The effect of siMIN inhibition on the MHV sgRNA synthesis was quantitatively analyzed with Image J software. (f) The one-step growth experiments of MHV-A59-infected DBT-3′siRNA cells treated with 20 nM siMIN. 5 × 10^4^ DBT cells were seeded in 24-well plates and infected with MHV-A59 at an M.O.I of 3 for 0, 6, 12, 18, and 24 h the next day. After each infection time, MHV-A59 was harvested and the growth rate of the virus was analyzed. (**g**) Representative plaque shape formed by MHV-A59 infecting DBT-3′siRNA cells treated with 20 nM siMIN. (**h**) Average plaque size of MHV-A59 formed on DBT-3′siRNA cells treated with 20 nM siMIN. Statistical analysis was performed using a student’s *t*-test and a one-way analysis of variance (ANOVA). A post-hoc test was conducted using Tukey’s multiple comparisons test. (**i**) Analysis of 3′UTR sequence of MHV-A59 infected DBT-3′siRNA cells treated with 20 nM siMIN. The MHV-A59 virus, harvested after infecting DBT-3′siRNA cells, was designated as P0. Subsequently, the harvested virus obtained after re-infecting cells was named P1, and the virus obtained after another round of infection and harvest was named P2. RNA was extracted from each of these virus samples, and sequence analysis was performed. (**j**) Detection of MHV-A59 gRNA in DBT cells treated with 10 nM siMIN. MHV-A59 negative-strand RNA and positive-strand gRNA were quantified relative to actin control by RT-qPCR assay. The negative-strand RNA synthesis in siMIN-treated DBT cells was statistically significantly decreased compared to the negative-strand RNA synthesis in siCon-treated DBT cells. Statistically significant groups were labeled as A and B to differentiate them. * indicates a statistical difference in the *t*-test between the shCon-treated plaque size group and the shSL1/2 plaque size group; ** indicates a statistical difference in the *t*-test between the shCon-treated plaque size group and the shSL3/4 plaque size group; *** indicates a statistical difference in the *t*-test statistical difference between the shCon-treated plque size group and the siMIN-treated plque size group is significant.

Subsequently, the RNA dot blot assay was employed to assess the inhibitory impact of siRNA targeting the MHV-A59 3′UTR. In order to achieve this, we infected DBT-3′siRNA cells (collectively named multiple DBT cells treated with siRNAs targeting the 3′UTR, Supplemental Table S2) with MHV-A59. Subsequently, total RNA was extracted from the aforementioned DBT-3′siRNA cells and subjected to the RNA dot blot assay. The results demonstrated the efficacy of siRNAs in inhibiting MHV-A59 replication in DBT cells (Figure 5a,b). The replication of MHV-A59 was reduced by 7% in DBT cells treated with 100 mM siHVR, but was reduced by 99.5% in DBT cells treated with 100 mM siMIN (Figure 5c).

In light of our previous findings that the MHV-A59 genome was significantly reduced in the RNA dot blot assay, we postulated that siMIN would also inhibit MHV-A59 sgRNA synthesis. To verify this hypothesis, a Northern blot assay was performed using a probe derived from the MHV-A59 *N* gene. The results indicated that siMIN reduced overall sgRNA synthesis by approximately 60%, with a specific reduction of mRNA2 synthesis by about 86% (Figure 5d,e).

Given that sgRNA synthesis was diminished, it was predicted that siMIN would impact the MHV-A59 life cycle. A single-step growth experiment on MHV-A59-infected DBT-3′siMIN cells demonstrated this, indicating that siMIN initiated a reduction in the viral growth rate at 18 h post-infection, with a 69% decrease in viral titer at 24 h post-infection compared to the control (Figure 5f).

Subsequently, the plaque sizes produced by MHV-A59 in DBT-3′siMIN cells were quantified after 24 h. The average plaque size in DBT-siCon cells was 1.59 mm, while in DBT-3′siMIN cells, it was reduced to 1.40 mm (Figure 5h), indicating a significant impact of siMIN on plaque formation.

As with our analysis of siSL1, we proceeded to investigate the progeny viruses resulting from a two-step sequential passage of MHV-A59 in DBT-3′siMIN cells. The progeny virus derived from the initial infection was designated P1_siMIN, while the progeny virus derived from the second infection was designated P2_siMIN. Sequencing the 3′UTR regions of these progeny viruses revealed that siMIN did not induce any genetic changes in the 3′UTR after the two-step sequential passage (Figure 5i). Similarly, sequencing of the 5′UTRs demonstrated that no genetic alterations had occurred (Supplemental Figure S2).

Finally, the impact of siMIN on gRNA synthesis was examined. Previous studies by Lai et al. identified the MIN structure as a cis-acting signal that is crucial for MHV DI negative-strand RNA synthesis [23]. Based on these observations, we postulated that siMIN would inhibit MHV-A59 negative-strand gRNA synthesis. The results demonstrated that 10 nM siMIN treatment significantly inhibited both negative- and positive-strand gRNA synthesis, with a more pronounced inhibition of the negative strand (Figure 5j).

In conclusion, the knockdown of the MIN structure by siMIN severely impairs MHV-A59 replication by inhibiting sgRNA synthesis, reducing viral growth rate, and affecting plaque size, without inducing genetic changes in the 5′ or 3′ UTRs.

### 3.6. The Combination of siRNAs Targeting the 5′UTR and 3′UTR Synergistically Inhibits MHV-A59 Replication

**Figure 6:**
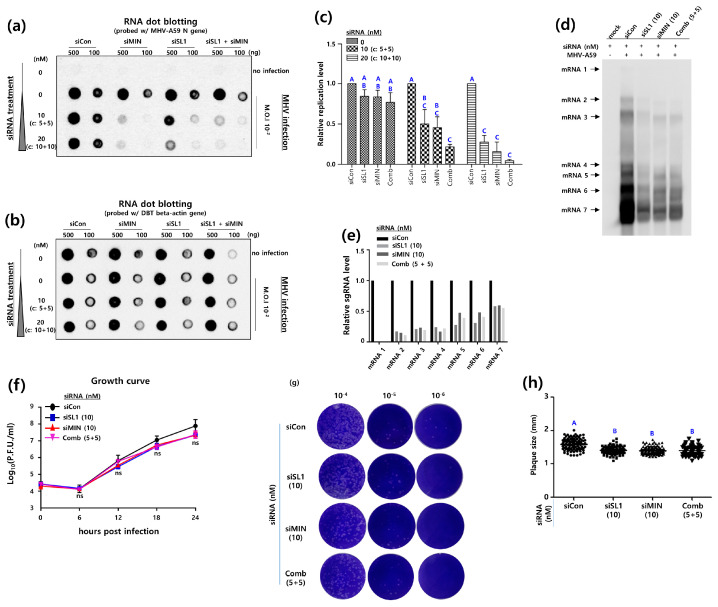
Inhibitory effects of siRNAs targeting SL structures in the 5′UTR and 3′UTR of MHV-A59. (**a**) RNA dot blot assay to analyze the effectiveness of the combination of siSL1 (5′siRNA) and siMIN (siRNA) to inhibit MHV-A59 replication. DBT cells (DBT-5′3′siRNA cells) were treated with two concentrations of siSL1 and siMIN combinations. For siSL1 and siMIN monotherapy, concentrations of 10 nM and 20 nM of each siRNA were used, respectively. For combination therapy, a half-and-half mixture of the two siRNAs at concentrations of 10 nM (5 nM siSL1 + 5 nM siMIN) and 20 nM (10 nM siSL1 + 10 nM siMIN) was used. DBT-5′3′siRNA cells were infected with MHV-A59 at an M.O.I of 10^−2^ for 24 h. RNA dot blot probes were made to detect the MHV *N* gene. (**b**) As an internal control, the expression of the actin gene in DBT-5′3′siRNA cells was analyzed by RNA dot blot assay. (**c**) The inhibitory effect of MHV-A59 replication by the combination therapy of siSL1 and siMIN (5 + 5, 10 + 10) was quantitatively analyzed by Image J software. MHV-A59 *N* gene expression was evaluated relative to actin gene expression in DBT-5′3′siRNA cells infected with MHV-A59. A primary one-way analysis of variance, followed by a secondary Tukey’s multiple comparison test was performed to determine the statistical significance between siSL1, siMIN, and the combination. Statistically significant groups were labeled as A, B and C to differentiate them. (**d**) Northern analysis for inhibitory effect of the combination therapy of siSL1 and siMIN (5 + 5) on the MHV sgRNA synthesis. MHV-A59 at an M.O.I of 0.5 was infected into DBT-5′3′siRNA cells for 24 h. Mock (negative control) was performed with DBT-5′3′siRNA cells that were not infected with MHV-A59. Patterns of MHV-A59 sgRNA production in DBT-5′3′siRNA cells were investigated with a DIG labelled *N* gene probe. (**e**) The inhibitory effect of the combination therapy of siSL1 and siMIN (5 + 5) on the MHV sgRNA synthesis was quantitatively analyzed with Image J software. Based on the sgRNA production of MHV-A59 infecting siCon-treated DBT cells, we quantified the sgRNA production of MHV-A59 infecting siSL1, si3MIN, and the combination-treated DBT cells. (**f**) The one-step growth experiments of MHV-A59-infected DBT-5′3′siRNA cells treated with the combination therapy of 5 nM siSL1 and 5 nM siMIN (5 + 5). 5 × 10^4^ DBT cells were seeded in 24-well plates and infected with MHV-A59 at an M.O.I of 3 for 0, 6, 12, 18, and 24 h the next day. After each infection time, MHV-A59 was harvested and the growth rate of the virus was analyzed. (**g**) Representative plaque shapes formed by MHV-A59 infecting DBT-5′3′siRNA cells treated with the combination therapy of 5 nM siSL1 and 5 nM siMIN (5 + 5). (**h**) Average plaque size of MHV-A59 formed on DBT-5′3′siRNA cells treated with the combination therapy of 5 nM siSL1 and 5 nM siMIN (5 + 5). Statistical analysis was performed using a student’s *t*-test and a one-way analysis of variance (ANOVA). A post-hoc test was conducted using Tukey’s multiple comparisons test. Statistically significant groups were labeled as A and B to differentiate them.

The objective of this study was to determine the efficacy of siRNA combinations targeting the 5′UTR and 3′UTR, respectively, in inhibiting MHV-A59 replication. The RNA dot blot assay was employed to investigate the impact of the combination of 5 nM siSL1 and 5 nM siMIN (5 + 5) and the combination of 10 nM siSL1 and 10 nM siMIN (10 + 10) on MHV-A59 total RNA synthesis (Figure 6a,b).

The replication of MHV-A59 was reduced by approximately 75.6% in DBT cells treated with a combination of 5 nM siSL1 and 5 nM siMIN (5 + 5) in comparison to the control. Similarly, MHV-A59 replication was reduced by 99.6% in DBT cells treated with a combination of 10 nM siSL1 and 10 nM siMIN (10 + 10) compared to the control. However, the inhibitory effects of the combination of siSL1 and siMIN (5 + 5, 10 + 10) were not statistically superior to the effects of siSL1 or siMIN alone (10 nM, 20 nM) (Figure 6c).

Given that the MHV-A59 genome was significantly reduced in the previous RNA dot blot assay, it was assumed that the combination of siSL1 and siMIN would also inhibit the MHV-A59 sgRNA synthesis. To verify this hypothesis, a Northern blot assay was performed using a probe derived from the MHV-A59 *N* gene. The impact of the siSL1 and si3MIN combination (5 + 5) on MHV-A59 sgRNA synthesis was investigated (Figure 6d). The production of MHV-A59 sgRNA was significantly impeded by the siSL1 and siMIN combination (5 + 5). However, the combination treatment (5 + 5) did not result in a greater inhibition of MHV-A59 sgRNA production than the 10 nM siSL1 and 10 nM siMIN alone (Figure 6e).

Given that the sgRNA synthesis was reduced in the Northern blot assay, it was postulated that the combination of siMIN and siSL1 would also affect the life cycle of MHV-A59. To verify this hypothesis, a single-step growth experiment was conducted on MHV-A59-infected DBT-5′3′siRNA cells (DBT cells treated with siSL1 and siMIN). The combination of siSL1 and siMIN (5 + 5) did not result in a statistically significant reduction in MHV-A59 titers compared to 10 nM siSL1 and 10 nM siMIN alone (Figure 6f). Furthermore, the sizes of plaques produced by MHV-A59 that infected DBT-5′3′siRNA cells for 24 h each were analyzed. Plaque sizes were analyzed by reinfecting L2 cells with the aforementioned MHV-A59 (Figure 6g,h). It is noteworthy that the mean plaque size of MHV-A59 in DBT-siCon cells was 1.59 mm, while that of MHV-A59 in DBT-3′siMIN cells was 1.39 mm. However, the combination of siSL1 and siMIN (5 + 5) was unable to form MHV-A59 plaques of a smaller size than those formed by 10 nM siSL1 and 10 nM siMIN alone (Figure 6h).

In conclusion, these results demonstrate that the combination of siSL1 and siMIN does not exhibit a synergistic effect on MHV-A59 replication, and that siSL1 and siMIN alone inhibit MHV-A59 replication to the same extent as the combination.

## 4. Discussion

The objective of this study was to ascertain whether an RNAi drug targeting the UTRs of MHV-A59 could effectively inhibit MHV-A59 replication. The UTRs are crucial components located at both ends of the MHV-A59 genome. They are known to be involved in replication, negative-strand gRNA synthesis, sgRNA synthesis, and virion assembly. Consequently, they represent a valuable target for the development of RNAi therapeutic agents against coronaviruses. Moreover, since the sgRNA synthesized by coronaviruses also contains the UTRs at both ends, a strategy targeting the UTRs offers the advantage of simultaneously targeting both the coronavirus gRNA and sgRNAs.

The results of our study indicate that RNAi targeting the SL1 structure in the 5′UTR is more effective in inhibiting MHV-A59 replication than RNAi targeting the MIN structure in the 3′UTR. Previous studies [18] have proposed a “dynamic SL1 model” in which the base of SL1 is optimized to mediate the physical interaction between the UTR and 3′UTR. This physical interaction was employed to stimulate MHV-A59 sgRNA production. The siSL1 targeted the base of SL1 and effectively inhibited MHV-A59 sgRNA production, whereas shSL1 targeted the loop of SL1 and failed to inhibit MHV-A59 replication. It is known that the 3′ end of the MHV-A59 genome is the site of initiation of negative-strand gRNA and sgRNA synthesis. Independent deletion analyses of MHV DI RNAs have identified a minimal site at the 3′ end of the genome that can support RNA synthesis, which has been named the MIN structure [21,25]. The siMIN, which targets the upstream loop and upstream stem of the MIN structure, has been demonstrated to have a very strong inhibitory effect on MHV-A59 replication, whereas 3′sh4, which targets the middle loop and downstream stem of the MIN structure, has been shown to have a minimal effect on MHV-A59 replication.

A portion of the *N* gene was employed as a probe to detect MHV-A59 replication. The relative analysis of the amount of MHV-A59 replication in this study was identical to the relative analysis of the production of *N* transcripts. From this perspective, the majority of the 5′shRNAs demonstrated a reduction in MHV-A59 replication in comparison to the control. The shSL1, shSL2, shSL3, and shSL4S were observed to have a relatively weak inhibitory effect on MHV-A59 replication, as they solely targeted the RNA secondary loop structure. However, shSL1/2, shSL3/4, and siSL1, which simultaneously targeted part of the loop and part of the stem, demonstrated relatively stronger inhibition of MHV-A59 replication. These findings indicated the potential existence of cis-acting elements crucial for MHV-A59 replication within the RNA secondary stem structure.

However, in contrast to RNAi, which targets the 5′UTR, the majority of 3′shRNAs (3′sh1, 3′sh2, 3′sh3, 3′sh4) demonstrated an increase in MHV-A59 replication. In particular, 3′sh3 and 3′sh4 enhanced MHV-A59 replication by approximately 12-fold compared to the control (Figure 4f). The sites targeted by 3′sh3 and 3′sh4 are the stem downstream of the 3′ MIN structure and the flanking loop. These sites are likely to be cis-acting elements that are deeply involved in MHV-A59 replication or sgRNA synthesis. Furthermore, 3′sh1 and 3′sh2 also demonstrated an increase in MHV-A59 replication, indicating that the pseudobases within the 3′UTR targeted by 3′sh1 and 3′sh2 may act as inhibitors of viral replication. Consequently, the knockdown of the pseudobases resulted in enhanced viral replication. Finally, it is possible that all three 3′shRNAs directly affected the MHV-A59 genome and altered the viral replication mechanism. Consequently, further studies are required to elucidate the mechanisms of action of these 3′shRNAs.

In single-step growth experiments designed to analyze the effect of RNAi on MHV-A59 growth, siSL1 and siMIN were observed to result in a decrease in virus titer and a slower rate of viral spread. It was postulated that the inhibitory effects of siSL1 and siMIN would result in the generation of distinct quasispecies of MHV-A59. Consequently, we conducted an examination of the genomic alterations in the 5′ untranslated region (UTR) and 3′UTR, which did not reveal any significant changes. Overcoming the replication inhibitory effect necessitates minor genome alterations, which are likely to induce various quasispecies, but major genome alterations are unlikely to induce quasispecies [29]. The absence of MHV-A59 quasispecies with genetic changes in UTRs in siSL1- and siMIN-treated DBT cells indicates that overcoming the inhibitory effects of siSL1 and siMIN requires major genomic changes. Nevertheless, although this study only examined genetic changes in UTRs, it is still possible that genetic changes in other parts of the MHV-A59 genome could occur. Further research is required to address this issue.

The single-step growth experience represents an experimental approach that permits a relative analysis of the titer and propagation rate of the virus [28]. The measurement of plaque size is crucial for comparative analysis of the viral propagation rate [28]. In general, if the plaque size is small, the single-step growth is often slowed down. The shSL1/2 and shSL3/4 have a growth curve that is 10 times slower than the control, and the average plaque size is also smaller than the control. Nevertheless, 3′sh1 exhibited a growth curve that was more than 10 times faster than the control, and the plaque size was also larger than the control.

SL1 is situated in close proximity to the 5′ extremity. SL1 exhibits low overall stability due to the presence of a high proportion of A-U and U-A base pairs. This low stability has been suggested to be important for MHV-A59 replication [18]. The siSL1 is thought to inhibit MHV-A59 replication by altering the low overall stability of SL1. The MIN structure was identified as a 3′ cis-acting signal for negative-strand RNA synthesis. It is postulated that siMIN inhibits MHV-A59 replication by inhibiting negative-strand RNA synthesis [21]. It is also known that SL1 and MIN structures interact with each other on the MHV-A59 genome.

According to a previous reference [18], the removal of A35 in the MHV-A59 5′UTR results in the conversion of 3′A29(31307) and 3′A78(31257) in the virus’s 3′UTR to 3′G29(31307) and 3′G78(31257), respectively. The 3′A29(31307)G change is located in the loop structure of the MIN. Based on this fact, the interaction of the SL1 structure containing 5′A35 with the MIN structure containing 3′A29(31307) was redesigned [18]. In other words, the interaction between the SL1 and MIN structures was interpreted as requiring specific base pairs at both sites. Consequently, the siSL1 and siMIN have the potential to disrupt the base pair interactions between the SL1 and MIN structures, which is thought to inhibit MHV-A59 replication [18]. Consequently, the siSL1 and siMIN combination is likely to act in a cooperative manner in inhibiting MHV-A59 replication. Nevertheless, no synergistic effect was observed between siSL1 and siMIN in inhibiting MHV-A59 replication. Although the two combinations did not demonstrate a statistically significant synergistic effect in this study, it is possible that a more precise measure of MHV-A59 replication inhibition may reveal a synergistic effect of siSL1 and siMIN.

MHV-A59 and SARS-CoV-2 are members of the same beta-coronavirus group and cause pathologically similar diseases in their hosts. This has led to studies utilizing MHV-A59 as an animal model for SARS-CoV-2. A comparison of the RNA secondary structures of the 5′ untranslated region (UTR) and 3′UTR reveals some unusual features. These include the presence of a hairpin loop in the 5′UTR and a pseudoknot in the 3′UTR. These features are discussed in detail in references, respectively [17,30]. Both viruses exhibit an SL1 structure in proximity to the extremity of the 5′UTR. However, MHV-A59 displays a more unstable SL1 structure than SARS-CoV-2. However, in the vicinity of the extremities of the 3′UTRs of both viruses, a MIN structure was identified in MHV-A59, but not in SARS-CoV-2. Consequently, the SL1 structure appears to be a universal structure present near the 5′ extremity of the majority of coronavirus genomes, while the MIN structure is a unique structure present near the 3′ extremity of the MHV-A59 genome. The strength of this study is that it provides a basis for the development of new drugs targeting RNA second SL structures of the 5′ and 3′ UTRs. However, the limitations are the lack of in vivo validation of the efficacy of targeted RNAi and the lack of a method to overcome systemic resistance. In vivo efficacy experiments of RNAi can utilize either liposome-mediated siRNA delivery or lentivirus-based shRNA delivery methods [31].

In conclusion, the findings of this study indicate that RNAi molecules targeting MHV-A59 5′UTR and 3′UTR are effective in suppressing MHV-A59 replication. In particular, SL1 and MIN structures are expected to be important molecular targets for RNAi drugs in the future when developing RNAi drugs to treat coronavirus infectious diseases.

## 5. Conclusions

In conclusion, this study identified the SL1 of the 5‘UTR and the minimal element for negative-strand RNA synthesis (MIN) of the 3‘UTR as potential targets for antiviral drugs against MHV-A59 using siRNA silencing. While the combination of siSL1 and siMIN did not show synergistic inhibition, SL1, conserved between MHV-A59 and SARS-CoV-2, represents a promising target for further development of RNAi therapeutics against SARS-CoV-2.

## Data Availability

The data presented in this study are available on request from the corresponding author.

## Supplementary Materials

The following supporting information can be downloaded at: https://www.mdpi.com/article/10.3390/pathogens13060518/s1, Figure S1: Types of DBT-5‘shRNA cells (a) and DBT-3‘shRNA cells (b) established in this study. Figure S2: Analysis of the 5′ and 3′ UTR sequences of MHV-A59 disrupted by RNAi targeting the MHV-A59 UTR SL structures. (a) Sequence analysis of the 3′ UTR of MHV-A59 targeted by shSL1/2 and shSL3/4. (b) Sequence analysis of the 3′ UTR of MHV-A59 targeted by siSL1. (c) Sequence analysis of the 5′ UTR of MHV-A59 targeted by siMIN. Table S1: List of RNAi oligonucleotides. Table S2: Names of DBT cells transfected with shRNA or treated with siRNA. Table S3: Primer list used in 5′ RACE and 3′ RACE assays.
