# Supplementary material for: Evaluation of RNA Secondary Stem-Loop Structures in the UTRs of Mouse Hepatitis Virus as New Therapeutic Targets
Source: Pathogens. 2024 Jun 19;13(6):518. doi: 10.3390/pathogens13060518 (PMC11206603; doi:10.3390/pathogens13060518)
Supplement: Supplementary file 1 [file pathogens-13-00518-s001.zip › pathogens-3021519-supplementary.pdf]

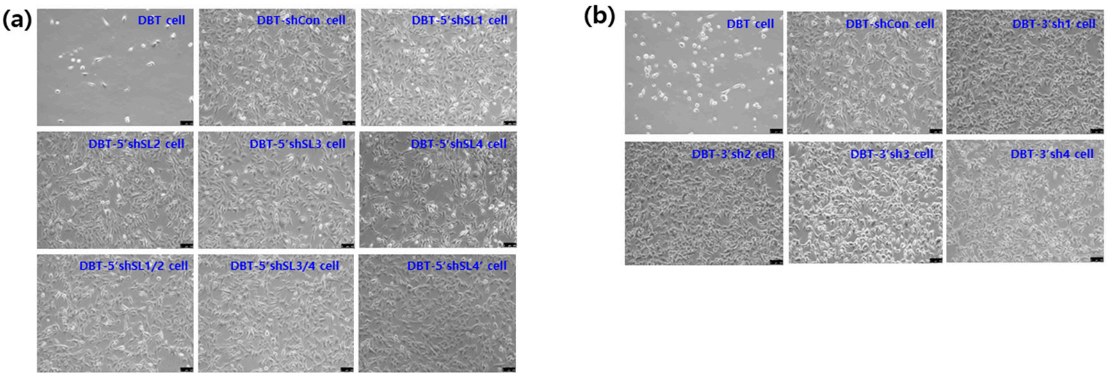

Figure S1. Types of DBT-5'shRNA cells (a) and DBT-3'shRNA cells (b) established in this study.

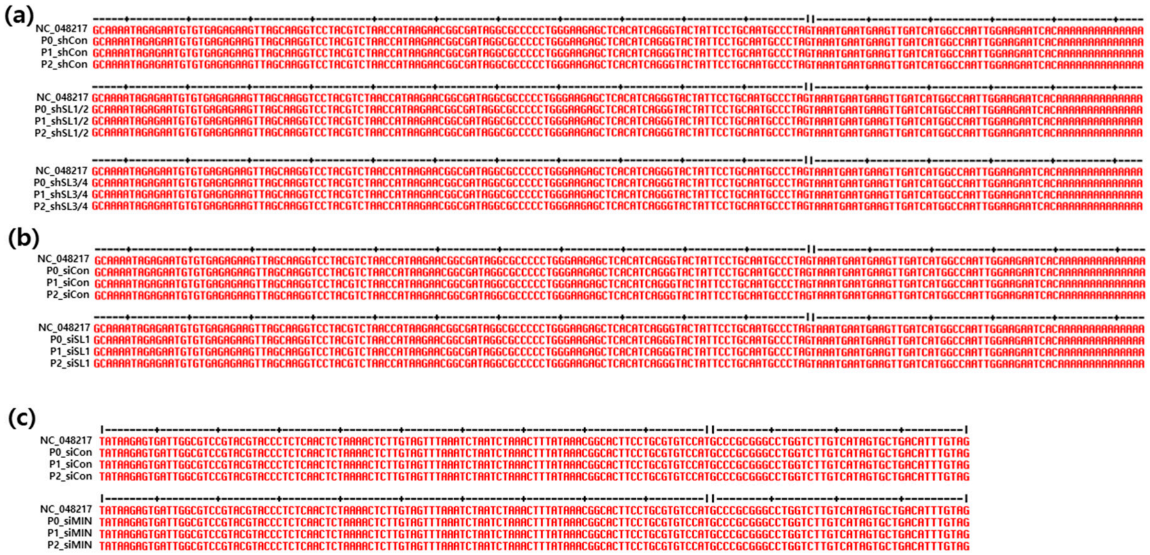

Figure S2. Analysis of the 5' and 3' UTR sequences of MHV-A59 disrupted by RNAi targeting the MHV-A59 UTR SL structures. (a) Sequence analysis of the 3' UTR of MHV-A59 targeted by shSL1/2 and shSL3/4. (b) Sequence analysis of the 3' UTR of MHV-A59 targeted by siSL1. (c) Sequence analysis of the 5' UTR of MHV-A59 targeted by siMIN.

Table S1. List of RNAi oligonucleotides.

Name	Sequence (5′ → 3′)	Location
shSL1 F	CCGGTCCGGGGCGTCCGTACGTACCCTCTCCTCTTCAAGAGAGAG GAGAGGGTACGTACGGACGCCTTTTTTGG	14~34
shSL1 R	AATTCCAAAAAAGGCGTCCGTACGTACCCTCTCCTCTCTCTTGAAG AGGAGAGGGTACGTACGGACGCCCCGGA	14~34
shSL2 F	CCGGTCCGGAAAACTCTTGTAGTTTAACTCTTCAAGAGAGAGTTA AACTACAAGAGTTTTTTTTTGG	41~58
shSL2 R	AATTCCAAAAAAAAAACTCTTGTAGTTTAACTCTCTCTTGAAGAGT TAAACTACAAGAGTTTTCCGGA	41~58
shSL3 F	CCGGTCCGGTCTAATCTAAACTTTATAAACCTCTTCAAGAGAGAGG TTTATAAAGTTTAGATTAGATTTTTTGG	60~80
shSL3 R	AATTCCAAAAAATCTAATCTAAACTTTATAAACCTCTCTCTTGAAG AGGTTTATAAAGTTTAGATTAGACCGGA	60~80
shSL4 F	CCGGTCCGGGTGTCCATGCCCGCGGGCCTGGTCTTCTCTTCAAGAG AGAGAAGACCAGGCCCGCGGGCATGGACACTTTTTTGG	93~118
shSL4 R	AATTCCAAAAAAGTGTCCATGCCCGCGGGCCTGGTCTTCTCTCTCT TGAAGAGAAGACCAGGCCCGCGGGCATGGACACCCGGA	93~118
shSL1/2 F	CCGGTCCGGACGTACCCTCTCAACTCTAAACTCTTCAAGAGAGAG TTTAGAGTTGAGAGGGTACGTTTTTTTGG	23~43
shSL1/2 R	AATTCCAAAAAAACGTACCCTCTCAACTCTAAACTCTCTCTTGAAG AGTTTAGAGTTGAGAGGGTACGTCCGGA	23~43
shSL3/4 F	CCGGTCCGGTAAACTTTATAAACGGCACTTCTCTTCAAGAGAGAG AAGTGCCGTTTATAAAGTTTATTTTTTGG	67~87
shSL3/4 R	AATTCCAAAAAATAAACTTTATAAACGGCACTTCTCTCTCTTGAAG AGAAGTGCCGTTTATAAAGTTTACCGGA	67~87
shSL4′ F	CCGGTCCGGGTCATAGTGCTGACATTTGTACTCTTCAAGAGAGAGT ACAAATGTCAGCACTATGACTTTTTTGG	119~139
shSL4′ R	AATTCCAAAAAAGTCATAGTGCTGACATTTGTACTCTCTCTTGAAG AGTACAAATGTCAGCACTATGACCCGGA	119~139
siSL1 (sense)	AAGAGUGAUUGGCGUCCGUACUU	5~24
siSL1 (antisense)	GUACGGACGCCAAUCACUCUUUU	5~24
siSL3/4 (sense)	AAACGGCACUUCCUGCGUGUCUU	77~97
siSL3/4 (antisense)	GACACGCAGGAAGUGCCGUUUUU	77~97
3′sh1 F	CCGGTCCGGATGGATGTCTTGCTGTCATAACTCTTCAAGAGAGAG TTATGACAGCAAGACATCCATTTTTTTGG	31,109~31,129
3′sh1 R	AATTCCAAAAAAATGGATGTCTTGCTGTCATAACTCTCTCTTGAAG AGTTATGACAGCAAGACATCCATCCGGA	31,109~31,129
3′sh2 F	CCGGTCCGGGTGGCAGACCCTGTATCAATTCTCTTCAAGAGAGAG AATTGATACAGGGTCTGCCACTTTTTTGG	31,145~31,163
3′sh2 R	AATTCCAAAAAAGTGGCAGACCCTGTATCAATTCTCTCTCTTGAA GAGAATTGATACAGGGTCTGCCACCCGGA	31,145~31,163
3′sh3 F	CCGGTCCGGAGCTCACATCAGGGTACTATTCTCTTCAAGAGAGAG AATAGTACCCTGATGTGAGCTTTTTTTGG	31,259~31,280
3′sh3 R	AATTCCAAAAAAAGCTCACATCAGGGTACTATTCTCTCTCTTGAA GAGAATAGTACCCTGATGTGAGCTCCGGA	31,259~31,280

3′sh4 F	CCGGTCCGGCCTGCAATGCCCTAGTAAATGCTCTTCAAGAGAGAG CATTTACTAGGGCATTGCAGGTTTTTTGG	31,281~31,301
3′sh4 R	AATTCCAAAAAACCTGCAATGCCCTAGTAAATGCTCTCTCTTGAA GAGCATTTACTAGGGCATTGCAGGCCGGA	31,281~31,301
siHVR (sense)	AACCAUAAGAACGGCGAUAGGUU	31,224~31,246
siHVR (antisense)	CCUAUCGCCGUUCUUAUGGUUUU	31,224~31,246
siMIN (sense)	AAUGAAGUUGAUCAUGGCCAAUU	31,301~31,323
siMIN (antisense)	UUGGCCAUGAUCAACUUCAUUUU	31,301~31,323

Table S2. Names of DBT cells transfected with shRNA or treated with siRNA.

Name of shRNA or siRNA	Methods to build or treat	Name of DBT cells
shControl vector	Transfected into DBT cells with lentiviral packing kit (shControl) and selected with puromycin	DBT-shCon cell
shSL1 vector	Transfected into DBT cells with lentiviral packing kit (shSL1) and selected with puromycin	DBT-5′shSL1 cell
shSL2 vector	Transfected into DBT cells with lentiviral packing kit (shSL2) and selected with puromycin	DBT-5′shSL2 cell
shSL3 vector	Transfected into DBT cells with lentiviral packing kit (shSL3) and selected with puromycin	DBT-5′shSL3 cell
shSL4 vector	Transfected into DBT cells with lentiviral packing kit (shSL4) and selected with puromycin	DBT-5′shSL4 cell
shSL1/2 vector	Transfected into DBT cells with lentiviral packing kit (shSL1/2) and selected with puromycin	DBT-5′shSL1/2 cell
shSL3/4 vector	Transfected into DBT cells with lentiviral packing kit (shSL3/4) and selected with puromycin	DBT-5′shSL3/4 cell
shSL4′ vector	Transfected into DBT cells with lentiviral packing kit (shSL4′) and selected with puromycin	DBT-5′SL4′ cell
3′sh1 vector	Transfected into DBT cells with lentiviral packing kit (3′sh1) and selected with puromycin	DBT-3′sh1 cell
3′sh2 vector	Transfected into DBT cells with lentiviral packing kit (3′sh2) and selected with puromycin	DBT-3′sh2 cell
3′sh3 vector	Transfected into DBT cells with lentiviral packing kit (3′sh3) and selected with puromycin	DBT-3′sh3 cell
3′sh4 vector	Transfected into DBT cells with lentiviral packing kit (3′sh4) and selected with puromycin	DBT-3′sh4 cell
siSL1	Transfected siRNA (siSL1) into DBT cells for 24 h using RNAiMAX reagent	DBT-5′siSL1 cell
siSL3/4	Transfected siRNA (siSL3/4) into DBT cells for 24 h using RNAiMAX reagent	DBT-5′siSL3/4 cell

siHVR	Transfected siRNA (siHVR) into DBT cells for 24 h using RNAiMAX reagent	DBT-3′siHVR cell
siMIN	Transfected siRNA (siMIN) into DBT cells for 24 h using RNAiMAX reagent	DBT-3′siMIN cell
siSL1 + siMIN	Transfected siRNA (siSL1, siMIN) into DBT cells for 24 h using RNAiMAX reagent	DBT-5′3′siRNA cell
5′shRNAs	Transfected into DBT cells with lentiviral packing kit (5′shRNAs) and selected with puromycin. Collectively named multiple DBT cells expressing shRNAs targeting the 5'UTR.	DBT-5′shRNA cell
3′shRNAs	Transfected into DBT cells with lentiviral packing kit (3′shRNAs) and selected with puromycin. Collectively named multiple DBT cells expressing shRNAs targeting the 3'UTR.	DBT-3′shRNA cell
5′siRNAs	Transfected siRNA (5′siRNAs) into DBT cells for 24 h using RNAiMAX reagent. Collectively named multiple DBT cells treated with siRNAs targeting the 5'UTR.	DBT-5′siRNA cell
3′siRNAs	Transfected siRNA (3′siRNAs) into DBT cells for 24 h using RNAiMAX reagent. Collectively named multiple DBT cells treated with siRNAs targeting the 3'UTR.	DBT-3′siRNA cell

Table S3. Primer list used in 5′ RACE and 3′ RACE assays.

Name	Sequence (5′ → 3′)	Location
5′ RACE_R1	AAAGGTGAAAGGCCACGT	649 - 666
5′ RACE_R2	CGCTTGGATGGCTTAACC	521 - 538
5′ RACE_R3	CAGACTGAACACAGCATTCC	413 - 432
AP(dt)17	GATCAGGACGTTCGTTTGAGTTTTTTTTTTTTTTTTT	-
3′ RACE_F1	CAGAAGGATGGTGGTGCAG	30,806 – 30,824
3′ RACE_F2	GCAAAGCCCAAAAGCTCTG	30,905 – 30,923
